# Empowering coaching in youth basketball: the balancing mechanism between coaching authority and athlete autonomy

**DOI:** 10.3389/fpsyg.2025.1678196

**Published:** 2026-05-19

**Authors:** Junmin Zhang, Yongfeng Liu, Xiaogang Zhang, Ruobing Chen, Guang Feng, Shuiyou Hu

**Affiliations:** 1 School of Sports Training, Chengdu Sports University, Chengdu, Sichuan, China; 2 School of Football Sports, Chengdu Sport University, Chengdu, Sichuan, China; 3 School of Physical Education, Chengdu Sport University, Chengdu, Sichuan, China

**Keywords:** athlete-centered approach, coaching philosophy, empowering coaching, grounded theory, youth basketball training

## Abstract

**Objective:**

In training practices, ingrained power dynamics between coaches and athletes often hinder the adoption of an “athlete-centered” approach, leading to diminished efficiency in sports training. Consequently, academia calls for scholars to reflect on coach-athlete power relations and explore coaching approaches aligned with athletes’ needs. In youth basketball training, how can coaches maintain authority while granting athletes’ autonomy and placing them at the core of training? However, this balance remains under-explored in the literature.

**Methods:**

This study adopted U. S. youth basketball training as a case, with primary data collected from publicly available coach interviews on the Spotify audio streaming platform. We systematically applied Procedural Grounded Theory (PGT) to analyze the transcribed content.

**Results:**

The study found that coaches’ identification with basketball in family, community, and professional environments generated strong affective organizational commitment, prompting a person-centered sense of responsibility and professional mission, which culminated in self-disciplined behavior. This commitment and responsibility enable coaches to form a coaching philosophy centered on the interconnection of team awareness, Balancing Rigor and Flexibility, and humanistic philosophy, which is manifested as scientific talent selection, developmental guidance, heuristic tactical guidance, and empowerment-oriented management during training, thereby achieving a dynamic balance between coaching authority and athlete autonomy.

**Conclusion:**

The theoretical model constructed in this study establishes a new theoretical framework for subsequent research and provides youth basketball coaches with practical and replicable coaching guidelines. It can help coaches implement the “athlete-centered” approach and enhance coaching effectiveness.

## 1 Introduction

Implementing the “athlete-centered” approach in sports training is recognized as the foundation of high-quality coaching (Bowles and O’Dwyer, 2020). However, adolescent athletes’ psychological needs often conflict with coaches’ instructional requirements. This conflict further complicates the implementation of the athlete-centered approach. Previous studies have shown that as adolescents begin to form self-awareness and desire autonomy (Spear and Kulbok, 2004), more authoritarian coaching styles may trigger dissatisfaction, undermining the coach-athlete relationship (Liu and Chen, 2024) and thus leading to disciplinary issues and resistance (De Laet et al., 2016). Conversely, establishing authority is a hallmark of effective coaching leadership (Zhang et al., 2024), often achieved through punitive measures and criticism to correct misbehavior (Kerr et al., 2020). In practice, youth basketball coaches struggle to balance the assertion of their authority with respect for athletes’ autonomy and individuality, limiting the educational effectiveness of youth basketball programs.

Accordingly, academia has called for scholars to explore humanistic coaching methods in coach-led environments (Nelson et al., 2014), reexamine the power relations between coaches and athletes, and develop coaching approaches more aligned with athletes’ needs (Denison et al., 2017). How can youth basketball coaches maintain authority while empowering athletes and placing them at the core of training? Answering this question would provide theoretical foundations for innovating coaching philosophies and enhancing the developmental outcomes of youth basketball programs.

## 2 Literature review

Coaching philosophy is a widely discussed concept in academia. Some scholars define coaching philosophy from a practical perspective as the ideological foundation guiding coaches to adopt optimal methods for enhancing athletes’ comprehensive capabilities (Collins et al., 2009). Others approach it from a values-based perspective, arguing that coaches’ personal values and beliefs influence their coaching practices, and that coaching philosophy is a coherent framework that organizes these values, beliefs, assumptions, attitudes, principles, and priorities (Nash et al., 2008; Carless and Douglas, 2011). However, other scholars have criticized these two perspectives, arguing that existing research neglects the influence of external environmental factors such as social, cultural, and ideological contexts on coaching philosophies, while overemphasizing coaches’ agency and dominance in sports training. They advocate that academia should explore the complexity and uncertainty that coaches face in practice when studying coaching philosophies (Cushion and Partington, 2016).

When scholars attempt to translate abstract philosophies into observable practice models, two coaching approaches with distinct value orientations have gradually emerged. Some scholars, advocating for the humanistic value of “achieving comprehensive human development,” have summarized an athlete-centered approach. This method requires coaches to implement humanistic principles, adhere to people-oriented concepts (Nelson et al., 2014), and emphasize guiding athletes to learn actively through questioning and discussion (Bowles and O’Dwyer, 2020). In contrast, other scholars inherit the pragmatic school’s pursuit of “optimal methods.” They argue that coaches should lead sports training and maintain authority, forming a coach-centered approach (Williams and Hodges, 2005). On this basis, scholars have analyzed the effects of these coaching methods and found that the athlete-centered approach can enhance athletes’ competitive performance, participation motivation, and positive learning experiences (Light and Harvey, 2017), while also contributing to the development of social–emotional skills such as cognitive ability, leadership, and psychological resilience (Cardia, 2024).

As an important link between abstract philosophies and coaching practice, coaching behaviors and leadership styles have become a major focus of academic research. Coaching behaviors are the concrete embodiments of coaching philosophies in training practices. Academically, these behaviors are commonly classified into several categories, including Training and Instruction, Democratic Behavior, Autocratic Behavior, Social Support, Positive Feedback, and Autonomy Support (Chelladurai and Saleh, 1980; Deci and Ryan, 1987). Studies have shown that coaches’ autonomy-supportive behaviors help establish positive coach-athlete relationships and reduce athletes’ burnout levels (Kim and Choi, 2024). While controlling behaviors can correct the bad habits of athletes (Delrue et al., 2019), they simultaneously have negative impacts on coach-athlete relationships and athletes’ motivation to participate in training (Liu and Chen, 2024).

Leadership styles refer to the behavioral patterns exhibited by leaders in guiding team members to achieve goals, which can be divided into democratic (Gastil, 1994), Autocratic (Ali, 2019), transformational (de Lopez Subijana et al., 2021), and ethical (Constandt et al., 2020). Further research on the effects of these leadership styles can be divided into two streams. One focuses on analyzing how different leadership styles influence external factors. For example, studies have confirmed that ethical leadership has positive effects on team moral climate (Constandt and Willem, 2019), athletes’ emotional organizational commitment, sense of responsibility, voice, and competitive performance (White and Rezania, 2019). The other stream focuses on how external factors influence coaching leadership styles. For instance, a study indicates that a sports club’s autonomy-supportive environment mediates the relationship between coaches’ self-transcendence values and transformational leadership (Castillo et al., 2018).

Existing research has explored the types and roles of coaching philosophies, behaviors, and leadership styles from multiple perspectives. However, it has overlooked differences across the sport disciplines. In practice, athletes in individual sports perceive significantly higher levels of coach-provided autonomy support than those in team sports (Van de Pol Pol et al., 2015), indicating complex relationships between sport characteristics, coaching behaviors, and athlete needs. As a prototypical team sport, youth basketball training requires athletes to rely on coaches for technical and tactical guidance while also developing autonomous decision-making abilities in real competitions. This combination creates a dual demand for coaching authority and athlete autonomy. However, the absence of clear guidance on how to achieve this balance has limited the effectiveness of youth basketball training. Therefore, this study aims to address this gap by proposing a theoretical framework designed to help coaches navigate the tension between authority and autonomy in youth basketball training.

## 3 Research design

### 3.1 Method

**Figure 1:**
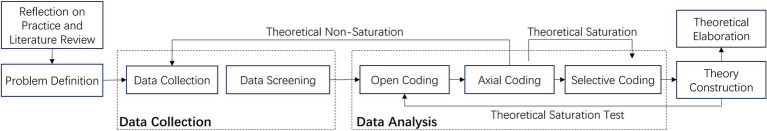
Coding process.

This study following the methodological norms of Procedural Grounded Theory (PGT), the inductive analysis of original materials proceeds through open coding, axial coding, and selective coding. In open coding, researchers analyze materials line-by-line to identify concepts within original sentences related to the research theme and group them into initial categories. Axial coding identifies master categories by examining relationships between initial categories. Selective coding establishes a core category linking all master categories, develops a coherent narrative, and constructs the theoretical model (see Figure 1).

### 3.2 Materials collection

This study adopted a purposive sampling approach. The primary data source was The Becoming, a podcast hosted on Spotify that focuses on basketball coaching and features interviews with experienced coaches and leaders in the sport. The selection of this podcast was based on its specific relevance to the research topic, as it provides a concentrated collection of professional coaching narratives.

A systematic screening process was applied to the podcast episodes, comprising a review of titles, an examination of descriptions, and preliminary listening. This process aimed to identify interview transcripts that were both highly relevant to the research topic and possessed substantial explanatory power. Data collection was discontinued when theoretical saturation was achieved, which was indicated by the fact that all emergent concepts and initial categories could be comprehensively encompassed by the core categories. Inclusion and exclusion criteria are shown in Table 1.

**Table 1:** Inclusion and exclusion criteria.

Inclusion criteria	Exclusion criteria
(1) The description of the interview record contained information pertinent to the research topic	(1) No description was available or if the description contained no information relevant to the research topic
(2) The interviewee was a youth basketball coach	(2) Individuals who were not youth basketball coaches
(3) The coach achieved strong competitive results or demonstrably cultivated elite athletes	(3) Despite a relevant description, the interviewee’s substantive responses diverged from the research topic

In total, the study included 13 coaches’ interview recordings, which were transcribed into over 150,000 Chinese characters of text materials (see Table 2).

**Table 2:** Demographic characteristics of interviewees.

Occupation	Gender	Key achievements
Head Coach	Male	Former National Collegiate Athletic Association (NCAA) Division III Basketball Player; Current Head Coach of a High School Basketball Team in Minnesota, USA
Head Coach	Male	Former Basketball Player; Current Head Coach of a High School Basketball Team.
Head Coach	Male	Has coaching experience in both high school and college basketball; Trained a player drafted by the NBA Mavericks
Head Coach	Male	Led teams to multiple high school tournament championships; Provided pre-draft training for NBA players
Head Coach	Male	Serves as head coach and talent scout for high schools, colleges, and National Strength and Conditioning Association (NSCA); Extensive coaching experience
Head Coach	Male	Long-term coaching experience in high school basketball
Head Coach	Male	Current Head Coach of a High School Boys’ Basketball Team in Texas; Previously coached at multiple renowned K-12 (Kindergarten through 12th grade) schools
Assistant Coach	Male	Current Basketball Skills and Performance Coach at a U. S. High School; Also serves as a College Basketball Assistant Coach
Head Coach	Male	USA Gold Coach; Head Coach of a High School Basketball Team
Community Coach	Male	D2 Youth Level Coach at Howell Basketball Club; Extensive coaching experience
Head Coach	Male	Coaches a top U. S. high school basketball program and serves as an assistant coach for multiple college teams
Head Coach	Male	Served as head coach for multiple U. S. high school basketball teams
Head Coach	Male	Former Basketball Player; Current Head Coach of a Junior High School Basketball Team

All audio data included in this study from Spotify is publicly accessible. This study adheres strictly to Spotify’s Privacy Policy and relevant ethical guidelines for secondary data use. According to Spotify’s terms and conditions, audio data on the platform may be used for data analysis and academic research, but only in a pseudo-anonymized format.

Ethical approval was obtained from the Ethics Committee of Chengdu Sport University (Approval No. CTYLL2025005).

## 4 Category extraction and model construction

### 4.1 Open coding

The transcribed raw data was imported into Microsoft Word. Initially, two researchers with professional basketball training and coaching experience conducted independent initial coding. Through a line-by-line reading of the transcripts, they identified statements of substantive significance to the research topic and conceptualized them. Subsequently, the two researchers jointly compared and discussed the initially derived concepts, documenting any discrepant codes. These concepts were then continually refined and revised throughout the subsequent coding process until a consensus was reached. Finally, this study identified 111 concepts and 27 initial categories. For example, “modeling effect,” “Value Inheritance,” and “Intergenerational career imitation” were grouped under “Family Support” as they relate to familial encouragement for coaches. Similarly, “public support” and “celebrity effect” were categorized as “Social Recognition” as they reflect societal validation of basketball coaching as a profession (Table 3).

**Table 3:** Example of open coding.

Initial categories	Concepts	Original sentence
Family support	Modeling effect	My father served as the head coach of the university team, and I love to watched him train when I was a child.
	Value inheritance	“Train for combat!” was the core philosophy he instilled in me, and I will strive to carry it forward.
Passion	Firm career commitment	My father wanted me to go to law school, but I defied his wishes and resolutely committed to a career as a basketball coach.
	Holistic engagement	Coaches must plan ahead, anticipate different response strategies, and commit themselves fully.
Autonomous dedication	Extra effort investment	Passion is the fuel for coaching. It drives me to invest extra effort and time to teach them.
	Prejudice elimination	High school coaches should collaborate with Amateur Athletic Union (AAU) team members. If they refuse to engage, you must take the initiative.
Earnestness	Stay focused	I try to keep myself as focused as possible, especially on the court.
	Fulfill the educational responsibility	Too many young coaches see coaching as just cool or fun. But that’s not true. There are responsibilities and obligations. You must truly help players improve.
Emotional interaction	Collective honor sense	Victory is not just a goal but an honor of fighting alongside teammates.
	Mutual trust	Everyone must trust that teammates will take on responsibilities to make the team better.
Upholding equality	Talent discrimination rejection	Even if you are not an athlete like Michael Jordan, you can strive to improve yourself.
	Athlete potential cultivation	Not everyone’s LeBron James. So, a coach has to help every player reach their full potential.
Encouraging independent decision-making	Fostering athlete self-confidence	It’s not just about chasing threes or inside shots. Athletes’ autonomous decisions are prioritized.
	Tactical mindset cultivation	During tactical training, we do not focus on strict step-by-step routines from A to B, but focus more on the conceptual level.
Educate through competitions	Combat first	The practiced techniques and tactics should be translated into practical competitive abilities through competitions.
	Against zone defense	I fully support the elimination of zone defense at the youth level! I think it is highly necessary to abolish zone defense during the youth stage.
Set basic behavioral boundaries	Management of daily conduct	At our university. No, players are not allowed to drink beer, man. This is a bit tricky.
	Balanced strictness and leniency	On the court, we are serious and no-nonsense. But off the court, everything is relaxed and joyful. We can crack jokes and enjoy the good times.
Implicitness	Selection of learning ability	The selection includes various tactical explanations. The goal is to test who can learn them in a short time, who can quickly recall and put them into action.
	Evaluate players’ desire to win	Even if you dislike teammates or your role, a desire to win lets you transcend all conflicts. Thus, recruiting players willing to win at all costs may matter most.

### 4.2 Axial coding

During the axial coding phase, we employed the constant comparative method to cluster interrelated initial categories, forming preliminary main categories. To ensure objectivity, two researchers who were not involved in the initial coding independently evaluated these results. A third researcher adjudicated any disagreements by referring to the original raw data. As a result, 10 master categories were developed from 27 initial categories. For example, “Family Support,” “Social Recognition,” and “Work environment empowerment” related to external environmental influences on coaching philosophies. Therefore, they were subsumed under “External Environment Immersion.” Coaching methods such as “Encouraging Independent Decision-Making,” “Emphasizing Awareness Training,” and “Acceptance of Athletes’ Mistakes” were employed to inspire tactical thinking and categorized under “Heuristic Tactical Guidance” (Table 4).

**Table 4:** Axial coding process.

Initial category	Main category
Family support, Family support, Work environment empowerment.	External environment immersion
Passion, Career satisfaction.	Affective organizational commitment
Career satisfaction, Active learning, Autonomous dedication.	Self-disciplined behavior
Earnestness, Optimism.	Balancing Rigor and Flexibility
Emotional interaction, Advocacy of team collaboration.	Team awareness
Acceptance of athletes’ mistakes, Upholding equality, Balancing training with academic learning.	Humanistic philosophy
Encourage independent decision-making, Emphasizing of awareness training, Acceptance of athletes’ mistakes.	Heuristic tactical guidance
Refine basketball skills, Educate through competitions, Inspire athletes to think proactively.	Developmental guidance
Set basic behavioral boundaries, Restraint by rules, Guidance for self-management.	Empowerment-oriented management
Implicitness, Interactivity, Systematic evaluation.	Scientific talent selection

### 4.3 Selective coding

Through mapping the relationships among master categories, we identified the core category of this study as “empowering coaching.” The theoretical narrative centered on this core category unfolds as follows.

**Figure 2:**
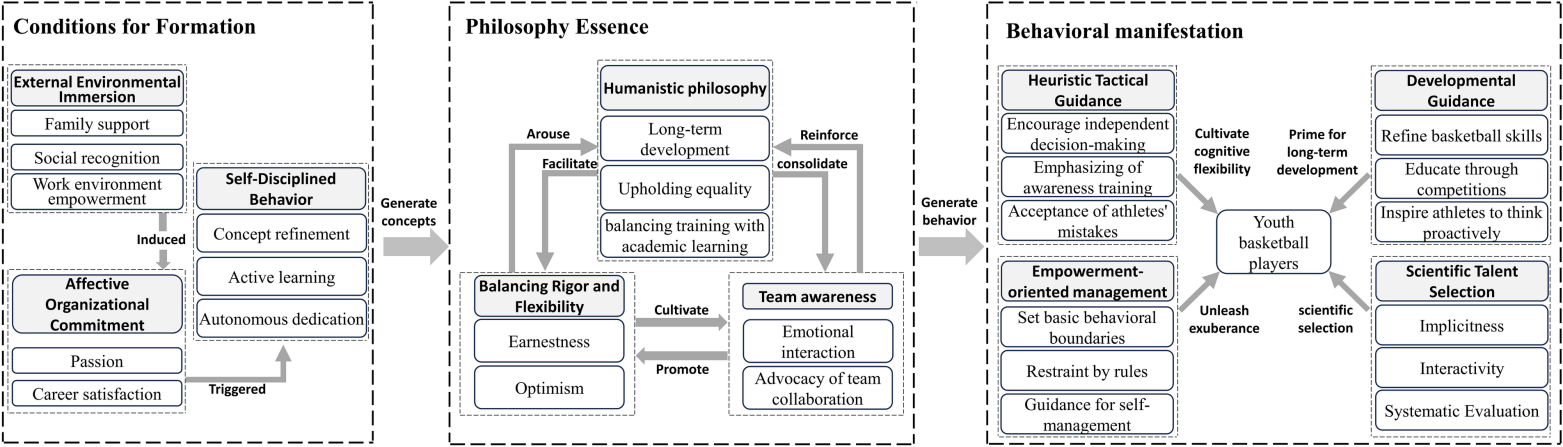
Empowerment-oriented coaching theory model.

Youth basketball coaches’ immersion in family support, social recognition, and work environment empowerment fosters affective organizational commitment, which in turn motivates self-disciplined behavior. This self-discipline process forms coaching ideologies structured around interconnected dimensions of team awareness, balancing rigor and flexibility, and humanistic philosophy, thereby giving rise to four types of coaching behavior, including scientific talent selection, developmental guidance, heuristic tactical guidance, and empowerment-oriented management. These practices balance coaching authority with athlete autonomy, facilitating comprehensive athlete development (Figure 2).

### 4.4 Theoretical saturation test

To maintain the integrity and validity of the research outcomes, this study adhered to the “multiple of six” criterion for theoretical saturation (Guest et al., 2006), supplementing the dataset with six additional online records from the Spotify platform. These data were systematically processed via open, axial, and selective coding, strictly following the established protocol. Notably, all newly generated concepts were fully subsumed within the existing master categories, and no novel relationships between categories were identified. Based on this, all researchers conducted group discussions to finalize the coding results and ensure the reliability of the study. This result indicates that the theoretical model illustrated in Figure 2 has achieved theoretical saturation.

## 5 Model interpretation

### 5.1 Formative conditions of empowerment-oriented coaching

#### 5.1.1 External environment immersion

In the United States, basketball is deeply embedded in the nation’s cultural and commercial systems (Sudre et al., 2019), providing a practical platform for cross-cultural communication among ethnic minorities (Thangaraj, 2010). This environment helps explain the popularity of basketball, laying a cultural foundation for youth basketball coaches to develop scientific coaching philosophies. Research indicates that parents’ passion for basketball can influence their children’s career choices through intergenerational transmission. As one interviewee stated: “I watched my father coach every day, and later even became one of his players. His coaching philosophy always fascinated me. My dream was to become a coach like him (C1).” For children, parental behaviors, attitudes, and educational approaches can serve as a primary model for exploring the world, potentially shaping their interests. Through long-term observation and imitation, the interviewee developed a strong vocational interest. This process led to gradually internalizing the coaching profession as a personal career ideal. This case illustrates the significant role of the role model effect in shaping vocational interests.

When these individuals become coaches, the culturally rich environment of American basketball may provide them with positive psychological feedback. This could foster their willingness to proactively collaborate with various stakeholders, including players, parents, and administrators, thereby strengthening their sense of professional identity. As another interviewee noted: “People always call me ‘Coach.’ I really like it, so the title just stuck (C5).” According to Social Cognitive Career Theory (SCCT), vocational interests form through personal participation, observational learning, and continual reception of external feedback. When individuals believe they are capable of performing a task and expect that the work will yield valuable outcomes, they are likely to develop a lasting interest in the activity, set corresponding goals, and gradually form firm career choices (Lent et al., 1994). The case of C5 supports the theoretical view of SCCT by illustrating the contribution of positive environmental feedback to strengthening a coach’s professional identity.

#### 5.1.2 Affective commitment

As discussed above, the professional identity formed through social interactions can foster a strong willingness to work among coaches. Existing research refers to this psychological mechanism as work passion, defined as the positive psychological energy and behavioral tendency individuals exhibit in vocational activities (Pollack et al., 2020). Scholars categorize it into obsessive passion and harmonious passion. The former occurs when an individual’s professional identity conflicts with their personal values and goals, leading to persistence driven by internal or external pressure, often resulting in emotional exhaustion. The latter refers to a state of psychological adaptation arising when a self-chosen professional identity aligns with one’s values and goals (Vallerand et al., 2003), leading to better job performance, higher levels of career satisfaction, and a more optimistic work mindset (Tolentino et al., 2022).

This study indicates that the continuous positive psychological feedback from the external environment satisfies coaches’ needs for competence and relatedness, which could prompt them to integrate personal values with their professional identity. This process may facilitate the formation and long-term maintenance of harmonious passion, potentially leading coaches to generate strong professional satisfaction. As expressed by an interviewee: “You will not see any of my achievements displayed, but the players know my contributions, and that’s enough for me. Day after day, I seek new methods to train athletes, helping them grow, learn, and improve. This brings me great happiness (C3).” According to Self-Determination Theory (SDT), the satisfaction of basic psychological needs motivates individuals to internalize the value and significance of a behavior, integrating external behavioral regulations with their own values (Deci and Ryan, 2008), thereby enhancing the persistence and effectiveness of their actions (Wangwongwiroj and Bumrabphan, 2021). The findings from this study are consistent with this view. In the case described, the coach gradually internalized helping athletes progress and grow as a core part of their self-worth, which can be interpreted as a transformation of an emotional connection initially based on interpersonal attachment into an active embrace of their professional mission, thereby forming an affective commitment to youth basketball coaching. At this stage, coaches no longer view athlete development and progress merely as a means to gain external validation. When faced with negative situations such as game losses or fluctuations in athletes’ performance, this sense of responsibility motivates them to actively analyze problems and seek solutions, thereby enhancing coaching efficacy.

#### 5.1.3 Self-disciplined behavior

The emotional attachment and value identification that US youth basketball coaches have with their profession is associated with a responsibility consciousness centered on “cultivating people.” In practice, this is reflected in additional work investment and proactive self-discipline regarding their own behaviors. As one interviewee stated: “Coaches who genuinely care about the kids’ development invest more energy in training and games, even using their rest time to learn new training methods, analyze game footage, etc. So, loving the sport and enjoying the process of helping children grow is more important than anything else. I will persist in doing this (C7).” According to existing research, this self-disciplined behavior supports the concept of a calling orientation to work. Individuals with a calling orientation typically deeply integrate the work itself with their personal values, and their work motivation stems primarily from internal identification rather than external controls (Wrzesniewski et al., 1997). In the case described, the coach’s behaviors exhibit characteristics that align with intrinsic motivation, potentially reinforced by the satisfaction derived from the coaching process. This behavioral pattern is fundamentally different from work orientations based on material rewards (job orientation) or career advancement (career orientation).

In summary, the “athlete-centered” approach is not an external requirement that needs to be learned and implemented, but rather a value belief consciously upheld by coaches to obtain positive psychological feedback. For coaches, maintaining their love for the sport and passion for coaching helps develop the intrinsic motivation for self-improvement and strict self-discipline. The principle of reciprocity in social exchange theory suggests that an individual’s act of goodwill often elicits a positive response from others (Cropanzano et al., 2017). Therefore, when athletes perceive the coach’s dedication and their own progress, they may become more cooperative, proactively seek help, and provide the coach with ample recognition and respect. This interactive pattern could strengthen the coach’s professional identity and sense of mission, making them more invested and self-disciplined in their work, potentially leading to a positive feedback loop.

### 5.2 The connotations of empowerment-oriented coaching

#### 5.2.1 Humanistic philosophy

By internalizing their professional role through social interactions, the coaches often adopt a humanistic orientation, centering on fostering the holistic development of individuals. In training, coaches maintain an egalitarian stance, refraining from discrimination based on athletes’ innate physical talent. They persistently explore the unique characteristics of each athlete’s competitive capabilities and employ various methods to unlock their developmental potential. As one interviewee stated: “No one is born as Michael Jordan. The role of a youth coach is to discover and ignite the potential within every player (C1).” It is crucial to note that the enhancement of athletic prowess is not an instantaneous process. Adolescent athletes exhibit significant individual variations in physical development, psychological maturity, and skill acquisition, leading to inherent performance variability (Rinaldo et al., 2020; Jawabreh et al., 2025). Coaches are thus required to approach youth basketball training as a long-term, coherent educational process, “designing targeted and scientific training regimens to ensure that the professional skills and theoretical knowledge acquired by athletes will benefit them throughout their lives (C9).” Thus, a defining characteristic of this coaching culture can be seen in the proactive identification and cultivation of strengths within each athlete’s competitive profile. This process involves utilizing scientifically-grounded and individualized training methods to help them develop distinctive skills. Under this philosophy, even athletes without exceptional physical gifts can gradually cultivate distinctive competitive advantages.

For athletes, sports training constitutes only one phase of life. Factors like injury and aging can terminate careers abruptly. To facilitate athletes’ successful career transitions, the coaches in this study often advocate for a talent development philosophy that balances academic and athletic pursuits. As an interviewee articulated: “Coaches must nurture various competencies in athletes, enabling them to make more composed career choices and achieve success in the future. The first lesson athletes need to learn is how to effectively balance their time between training and studies (C4).” For adolescents, engaging with theoretical knowledge not only sharpens logical thinking and social adaptation skills but also deepens their comprehension of basketball. Conversely, participation in sports training can alleviate psychological stress, enhance academic efficiency, and promote physical well-being. These two domains can be mutually reinforcing. In the case cited, the coach operates from a long-term educational perspective and designs basketball training content that promotes the synergistic development of athletes’ competitive abilities and comprehensive qualities, thereby granting them greater agency over their future lives. This approach exemplifies the humanistic philosophy within coaching practice.

#### 5.2.2 Team awareness

Team awareness refers to the collaboratively developed shared understanding among team members regarding game situations, tactical coordination, and related aspects (Bourbousson et al., 2015). In basketball, opportunities emerge with suddenness, complexity, and transience, necessitating seamless cooperation among players to be capitalized upon effectively. Consequently, well-developed team awareness is often considered an important factor for success in basketball. As one interviewee remarked: “When players communicate promptly, share ball possession, and strive to select the most opportune moments to attack during a game, they are playing the correct way (C7).” Through communication and collaboration, players continuously refine their in-game decision-making. This demonstrates the importance of team awareness in elevating overall team performance. However, this ideal collaborative state is not spontaneously achieved. In practice, “players instinctively focus on their personal scoring statistics during games (C9).” This pursuit of individual accolades often conflicts with the collective interests of the team. In such instances, if a coach mandates ball-sharing, it may foster a controlling coaching climate, which could provoke player resistance and undermine collaborative efficacy. According to Self-Determination Theory, the fundamental variation in individual motivation stems from differences in the degree to which the value of an activity has been internalized and integrated (Deci and Ryan, 2008). This suggests an association between the enhancement of teamwork efficacy and the extent of the athletes’ value identification with the collaborative process.

To guide athletes in integrating personal and collective interests, thereby stimulating their intrinsic motivation for proactive collaboration, American youth basketball coaches emphasize the cultivation of a sense of team belonging and collective honor. As interviewees described: “I organize gatherings with my players at my apartment, where we play games or go swimming. We hold such activities monthly. This fosters collective growth and strengthens team cohesion (C3). When players possess a strong willingness to cooperate with their teammates, we can achieve truly efficient collaboration in training and games (C6).” During competition, athletes driven by such intrinsic motivation are more likely to prioritize team success over personal statistics, strive to analyze game dynamics more rationally from a collective standpoint, and endeavor to make sound choices. To ensure effective teamwork, athletes also willingly align with the coach’s guidance, consciously acknowledging the coach’s leadership role. Thus, by encouraging active collaboration, mutual support, and proactively mediating team relationships, coaches may contribute to the development of players’ tactical awareness and help maintain a productive balance between coaching authority and athlete autonomy.

#### 5.2.3 Balancing rigor and flexibility

Balancing rigor and flexibility represents a core manifestation of coaching artistry. “Rigor” denotes the strict management coaches implement to achieve predefined training objectives. “Flexibility” refers to the tolerant atmosphere coaches create to stimulate athletes’ training enthusiasm. In youth basketball, excessive strictness can instill fear in athletes, eroding their confidence and creativity, while excessive leniency may lead to disorganized training, impeding the rate of skill development. Therefore, coaches must judiciously balance the degree of strictness and relaxation, taking into account factors such as athletes’ personalities, emotional states, and specific training contexts. The investigation revealed that coaches’ adept management of this balance relies not on external impositions but on their proactive modulation of coaching attitudes. Specifically, the coaching attitudes observed can be categorized into two dimensions: Earnestness and Optimism.

Earnestness refers to an individual’s focused and dedicated attitude toward a task and its goals. The study indicated that coaches who demonstrate a conscientious and dedicated attitude may help guide athletes toward developing self-disciplined habits, which could enhance training efficiency. As one interviewee noted: “The coach must approach the work with seriousness and use this attitude to inspire the entire team. Even in the absence of supervision, players will proactively engage in correct practices. This is the result of the coach’s conscientious and focused approach to coaching (C9).” Aligned with Social Learning Theory, individuals learn and develop through interactive processes with others (Bandura and Walters, 1977). This is also the case in the context of sports training. During training, athletes tend to observe and emulate the coach’s professional attitude. This process of observational learning can lead to the development of more self-disciplined behaviors. Unlike controlling coaching behaviors (e.g., physical punishment, reprimands, intimidation), earnestness can be described as a psychological state driven by an internal sense of responsibility. Its influence operates not through overt actions but through the powerful, subtle example set by wholehearted dedication to the coaching role.

Optimism is a psychological disposition wherein individuals focus on and emphasize the positive aspects of situations (Jackson, 2009). When confronting negative events such as defeats or performance errors, the coach’s timely error correction and patient explanation of better strategies exemplify this optimistic attitude. As an interviewee expressed: “Perhaps the athletes are performing poorly now, but they might suddenly grasp a technique during a future training session or game, leading to rapid performance improvement. Therefore, the coach’s role is to focus on the present and provide timely corrections (C7).” In this case, the coach reframes negative events like losses as opportunities for athlete growth. This approach is designed to safeguard the athletes’ self-efficacy, redirecting their focus from the fear of failure to the anticipation of skill enhancement. These conditions can foster more proactive and positive engagement in training.

### 5.3 Behavioral manifestations of empowerment-oriented coaching

#### 5.3.1 Scientific talent selection

Scientific talent selection refers to coaching behavior that involves a comprehensive evaluation of athletes’ implicit and explicit characteristics through a combination of quantitative assessment and long-term observation. Specifically, this behavior is primarily characterized by three aspects: implicitness, interactivity, and systematicity.

Implicitness denotes the coach’s emphasis on identifying and assessing latent traits in athletes, such as learning capacity and personality traits, during the selection process. As one interviewee stated: “During selection, I provide tactical explanations to test who can learn quickly, who can recall and apply knowledge promptly, thereby evaluating the athlete’s learning ability (C9).” It is important to note that whereas shortcomings in an athlete’s physical attributes (e.g., height, weight, Achilles tendon length) can be compensated for by developing technical-tactical skills or physical conditioning, they are rarely the sole determinants of competitive success. In contrast, implicit characteristics like personality, learning ability, competitive drive, and passion for basketball are less amenable to change through short-term external interventions. Therefore, these implicit characteristics should be incorporated into the evaluation criteria during the talent selection process.

Interactivity refers to the coach arranging interactions between team members and candidate athletes to scientifically assess their collaborative skills and team awareness. As an interviewee explained: “Selection is not just about choosing ability, but also about choosing partners. So, I have new players run drills with completely unfamiliar teammates and organize group discussions, all to see how quickly they can integrate into the team (C10).” Efficient collaboration among team members is a key factor for success in basketball. In this case, the coach’s proactive evaluation of an athlete’s capacity for teamwork can contribute to the development of a cohesive and hardworking team culture.

Systematicity means the coach comprehensively evaluates both the athlete’s short-term performance and long-term developmental potential. When selecting talents, coaches should not rely solely on subjective experience and intuition but should also employ scientific data analysis tools to systematically evaluate young athletes’ physical attributes and on-court performance. This can help coaches better understand the athlete’s competitive strengths, personality traits, and physical development, potentially laying the groundwork for targeted and scientifically grounded subsequent training plans. In summary, by employing diverse methods to comprehensively assess both implicit and explicit characteristics, coaches can help mitigate the risk of overlooking talent, thereby enhancing the effectiveness of the talent selection process.

#### 5.3.2 Developmental training instruction

Developmental training instruction refers to coaching behavior grounded in long-term educational planning, where training content is structured in stages according to the physical and psychological development patterns of youth athletes. The investigation found that the interviewed coaches typically regard mastering fundamental skills, optimizing offensive and defensive decision-making, and fostering independent thinking as the core tasks of youth basketball training, aiming to establish a solid foundation for the athletes’ long-term career development.

Influenced by internal and external factors such as learning capacity and training experience, youth athletes’ understanding of technical nuances is still developing, and the application of techniques in game situations has not yet been automated. Therefore, the coaches’ core responsibility in “building the foundation” is to help them refine technical details and understand the timing for applying various skills under high-intensity conditions. As an interviewee remarked: “In youth basketball games, use less half-court zone defense and employ more man-to-man defense. Allowing athletes to apply techniques in high-intensity confrontation effectively enhances their skill level (C3, C5).” Although flexibly using a combination of zone and man-to-man defense might be more conducive to achieving immediate competitive results, zone defense over-relies on rotational help from teammates and involves less physical contact. In the case above, the coach’s advocacy for man-to-man defense encourages athletes to apply techniques more frequently, which may contribute to improving their skill level. This approach aligns with the educational purpose of serving athlete development in youth competition.

“Give a man a fish and you feed him for a day; teach a man to fish and you feed him for a lifetime.” To foster athletes’ long-term development, youth coaches must not only help them build solid fundamental skills but also guide them to develop the habit of independent thinking, preparing them for higher competitive demands in the future. As one interviewee described: “I often have players review game footage, encourage them to express their ideas, make training suggestions, and I design training plans based on this input (C11).” On one hand, when coaches organize athletes to review and reflect on shortcomings identified in games, it helps them develop systematic and mature tactical awareness more quickly and improves their in-game decisions. On the other hand, by communicating with athletes as equals, transforming them from “decision-followers” to “decision-participants,” coaches aim to satisfy the athletes’ basic psychological needs, which could foster intrinsic motivation for independent thinking and dedicated training. When coaches incorporate athletes’ suggestions and ideas into scientifically designed training plans based on their professional expertise and experience, it may lead athletes to recognize the value of the coach’s targeted guidance, which could enhance their proactive communication. In summary, coaches who prioritize long-term athlete development over short-term competitive gains aim to create a virtuous cycle of “refining details, practical application, and reflective review.” This approach is consistent with the developmental characteristics of youth athletes, and has the potential to not only enhance their technical-tactical abilities but also foster independent thinking habits and strengthen their relationship with the coach, thereby contributing to improved efficiency of talent development.

#### 5.3.3 Heuristic tactical guidance

Heuristic tactical guidance is a coaching behavior that involves encouraging autonomous decision-making after the establishment of basic tactical frameworks, by providing timely correction and guidance. The investigation found that most interviewed coaches do not restrict athletes’ in-game decision-making during instruction but instead focus on cultivating their adaptability and independent decision-making skills. As one interviewee stated: “Before the game starts, I only outline the basic gameplay. I rarely implement fixed patterns from point A to B to C; instead, I encourage them to choose their offensive methods freely (C10).” This behavior aims to balance the coach’s authoritative guidance during games with the athletes’ autonomy and creativity. On one hand, youth athletes’ abilities to anticipate, seize, and adapt to offensive opportunities are still developing. These skills must be honed through repeated trial and error in competitive environments. Encouraging autonomous decision-making is intended to allow athletes to personally experience the specific timing and techniques for applying skills in game situations. This process encourages them to actively reflect on and summarize their experiences, which may deepen their tactical understanding. On the other hand, the coach’s prior establishment of basic tactical frameworks sets clear boundaries for in-game decisions, ensuring they remain within a reasonable tactical structure. When athletes make errors, the coach provides timely encouragement and explains specific improvement methods, which is designed to preserve the athletes’ enthusiasm for autonomous exploration while providing clear direction for their learning.

Specifically, the basic tactical approaches emphasized by the interviewed coaches during games can be categorized into three types: Quick decision-making, ball sharing, and adaptive response. Quick decision-making requires athletes to make prompt offensive and defensive choices based on intuition and perception. It is important to note that opportunities in basketball are fleeting, requiring athletes to anticipate based on the positioning of teammates and opponents. However, youth athletes’ capacity to “read the game” is underdeveloped, making it difficult for them to accurately anticipate and capitalize on emerging opportunities. This practice can help them to understand the timing and rhythm of actions, to expose flaws for correction in their decision-making process, and to potentially enhance the rationality and effectiveness of their choices. Ball sharing requires athletes to flexibly use various techniques while striving to find the optimal scoring opportunity. In youth basketball, excessive one-on-one play is a common issue. Instructing athletes to share the ball can help them recognize the importance of teamwork, better understand spatial relationships among teammates, and establish sound tactical thinking. Adaptive response requires athletes to appropriately apply techniques and tactics according to the evolving game situation. Due to limited experience, youth athletes often become passive when facing defensive adjustments. Therefore, guiding athletes to think proactively based on the game flow can enhance their tactical awareness and improve their decision-making quality.

#### 5.3.4 Empowerment-oriented management

Empowering management refers to coaching behavior where the coach establishes management rules equally binding on all team members, grants athletes self-management responsibilities, and facilitates peer monitoring. The investigation suggested that a system where both coaches and athletes adhere to the same rules helps mitigate the negative impacts of controlling behaviors. As an interviewee described: “In training, we have clear rules and discipline. For example, we call ‘holding the brick’ a ‘reminder of discipline.’ If an athlete makes a mistake, they have to ‘hold the brick.’ Actually, I’ve also held the brick because coaches make mistakes too. This is part of sports; the coach must lead by example (C10).” Although academia generally suggests that coaches’ controlling behaviors and the ego climate they create can struggle to satisfy athletes’ basic psychological needs and hinder the internalization of motivation (Sevil-Serrano et al., 2021), these studies often fail to analyze such behaviors within the dynamic context of coach-athlete interaction. According to standard research interpretations, “holding the brick” could be viewed as a physical punishment, potentially causing resistance. However, in the aforementioned case, by voluntarily submitting to the same team rules, the coach equalized their role and established a cohesive and equitable management climate. This climate is intended to satisfy athletes’ needs for belonging, which could counteract some negative effects associated with such disciplinary actions.

Once athletes develop team identification under the constraint of equal rules, this identification can support the internalization of team management regulations as necessary for achieving self-worth or team goals, moving beyond viewing them solely as external controls. At this point, with appropriate guidance, the aim is to stimulate athletes’ initiative for self-management, encouraging them to develop self-disciplined habits. As an interviewee stated: “I emphasize the importance of consistently maintaining good physical condition for training. Although I cannot monitor them constantly, if they truly love basketball and want to benefit from training, they will manage themselves effectively (C12).” By highlighting the value of self-disciplined behavior, the coach seeks to enhance athletes’ recognition of the causal relationship between poor choices and negative outcomes. This understanding is expected to encourage athletes to consider potential consequences before acting, proactively avoid inappropriate behaviors, and choose actions most likely to achieve their objectives. In summary, the essence of empowering management is to return the responsibility for growth to the athletes themselves, guiding them to develop self-disciplined habits, which helps lay the groundwork for the long-term development of their competitive abilities.

## 6 Practical implications

In basketball competitions, offensive or defensive opportunities are fleeting, requiring athletes to possess strong observational, judgment, and independent decision-making abilities—all closely tied to training. As leaders in sports training, coaches must balance maintaining their authority with granting athlete autonomy, avoiding inadvertently displaying controlling behaviors, physical punishment, or even emotional abuse toward athletes, thereby fostering athletes’ development.

This study found that the formation of an empowerment-oriented coaching philosophy depends on a positive feedback loop driven by external recognition. Therefore, sports management departments should promptly establish a performance evaluation system for youth sports coaches and conduct regular hierarchical evaluations, and establish corresponding coaching incentive mechanisms, offering rewards to coaches for outstanding contributions. With coaches’ consent, such departments or organizations should invite them to share coaching experiences via short videos, tweets, and other formats on major social media platforms. This will cultivate a positive public opinion environment for the coaching community and guide coaches to form a constructive competitive climate. Such external recognition will inspire coaches’ passion for coaching, prompting them to internalize athletes’ growth as validation of their own value, thereby laying the foundation for implementing empowerment-oriented coaching in training.

At the conceptual level, coaches must translate this philosophy into practice by serving as role models. This requires acknowledging that individual differences exist in adolescents’ physiological and psychological development cycles, with their competitive levels in sports training also varying. Coaches should not focus solely on athletes who currently perform well while neglecting those with significant developmental potential who have yet to demonstrate outstanding performance, in pursuit of competitive outcomes. Meanwhile, coaches should adhere to the coaching principles of equality, long-term development, and balancing training with academic learning; maintain a positive and optimistic attitude; encourage athletes to express their ideas; proactively communicate with them; and foster an equal, friendly, and positive team climate. As noted earlier, this not only highlights the exemplary role of coaches’ behaviors, guides athletes to develop positive training attitudes, and promotes the internalization of their training motivation but also lays a solid foundation for coach-athlete cooperation.

Finally, the implementation of this philosophy entails a set of concrete behaviors across the entire coaching process. Regarding talent selection, coaches need to focus on athletes’ physiological indicators and implicit traits, such as love for basketball, desire to win, and learning ability. Coaches should also conduct systematic evaluations of athletes’ developmental potential by analyzing game data and observing training and competition performance. In training, youth sports coaches should adhere to fostering athletes’ long-term development, refine basic technical and tactical training, and treat competitions as a training method. During competitions, coaches should formulate appropriate game goals based on athletes’ recent training, such as “focus on man-to-man defense, prioritize defending the opponent’s key players, or emphasize inside offense.” On this basis, coaches should encourage athletes to make independent decisions without strictly restricting offensive methods, guiding them to perceive in-game situational changes and thus develop systematic tactical awareness. After competitions, coaches should guide athletes to think proactively by reviewing game videos and writing competition summaries, enabling them to truly understand the spatial positions and movement purposes of both offense and defense, clarify the timing and rhythm of technical and tactical application, and become basketball talents who “truly understand training.” For team management, rules binding on both coaches and athletes should be established, with coaches managing athletes through such rules. Furthermore, a balance between strictness and leniency should be struck, providing athletes with leisure space to alleviate competitive pressure, inspire their love for training, and guide them to develop self-disciplined living habits.

## 7 Conclusion

Through systematic analysis of the raw data, this study constructed a theoretical model of Empowerment-Oriented Coaching, comprising three dimensions: Formative conditions, Philosophy essence, and Behavioral manifestations. The main conclusions are as follows.

(1) Through positive social interactions, coaches develop a strong professional identity by internalizing athlete development as core to their self-worth. This process fosters a sense of responsibility centered on “cultivating people,” indicating that the “athlete-centered” approach should not be an external mandate to be learned and implemented, but rather a value belief consciously upheld by coaches to obtain positive psychological feedback.

(2) In the context of youth basketball training, coaches should relinquish the pursuit of short-term competitive outcomes and instead position themselves as coordinators of team relationships, foundational builders of career development, and motivators of intrinsic drive. The core objective should be fostering athlete growth, thereby laying a solid foundation for their long-term athletic development.

(3) By adhering to an “athlete-centered” approach, coaches exhibit the following four categories of behavior: scientifically evaluating athletes’ developmental potential during talent selection to reduce the risk of missing potential talent; basing training on long-term developmental plans and scientifically designing staged training content according to the youths’ developmental characteristics; encouraging autonomous decision-making and providing timely correction and guidance during competitions, and cultivating self-disciplined lifestyle habits in daily life.

This study provides a concrete implementation framework for coaches to operationalize the “athlete-centered” approach, offering valuable insights for the transformation of coaching practices.

## 8 Limitation

This study was based on a sample of youth basketball coaches in the United States and did not explore differences in basketball culture and popularity across countries. Consequently, the theoretical model may lack applicability within the youth training systems and cultural contexts of other nations. Future research should incorporate diverse national contexts to optimize the model of empowering coaching behaviors, with the ultimate aim of supporting local coaches in innovating their philosophies.

Second, this study focused solely on examining the generative mechanisms of empowering coaching behaviors and did not assess their practical effects on coaching efficacy or coach-athlete relationships. Investigating these outcomes represents a critical direction for future research.

Finally, this study relied on secondary data, which may be susceptible to social desirability bias. That is, the interviewed coaches may have over-reported “athlete-centered” idealized methods to present a favorable image. Although we adhered to PGT norms and achieved theoretical saturation, the interpretation of the findings could still be influenced by this bias and the constraints of secondary data. Thus, future studies could adopt the Delphi method, engaging coaches in multiple validation rounds to refine the empowering coaching model.

## Data Availability

The raw data supporting the conclusions of this article will be made available by the authors, without undue reservation.
